# A Free-Standing Chitosan Membrane Prepared by the Vibration-Assisted Solvent Casting Method

**DOI:** 10.3390/mi14071419

**Published:** 2023-07-14

**Authors:** Urte Cigane, Arvydas Palevicius, Giedrius Janusas

**Affiliations:** Faculty of Mechanical Engineering and Design, Kaunas University of Technology, Studentu Street 56, 51424 Kaunas, Lithuania; giedrius.janusas@ktu.lt

**Keywords:** chitosan membrane, nanoporous AAO template, high-frequency excitation, two-step anodization method, solvent casting method, surface area

## Abstract

Much attention has been paid to the surface modification of artificial skin barriers for the treatment of skin tissue damage. Chitosan is one of the natural materials that could be characterized by its biocompatibility. A number of methods for the preparation of chitosan membranes have been described in scientific articles, including solvent casting methods. This study investigates an improved technology to produce chitosan membranes. Thus, chitosan membranes were prepared using a vibration-assisted solvent casting method. First, aqueous acetic acid was used to pretreat chitosan. Then, free-standing chitosan membranes were prepared by solvent casting on nanoporous anodized aluminum oxide (AAO) membrane templates, allowing for the solvent to evaporate. Using finite element methods, a study was obtained showing the influence of chitosan solutions of different concentrations on the fluid flow into nanopores using high-frequency excitation. The height of the nanopillars and the surface area of the chitosan membrane were also evaluated. In this study, the surface area of the chitosan membrane was found to increase by 15, 10 and 6 times compared to the original flat surface area. The newly produced nanopillared chitosan membranes will be applicable in the fabrication of skin barriers due to the longer nanopillars on their surface and the larger surface area.

## 1. Introduction

Skin is a human organ that acts as a physical barrier against external damage [1]. However, injuries can easily deform the multilayered structure and complications such as infections, melanoma, or the formation of scar tissue may result from the wound [2]. To treat wounds, artificial skin barriers could be applied [3]. These barriers can support cellular activity in a moist environment, allow for the transport of gaseous species, and simultaneously prevent the transmission of pathogens [4].

To create artificial skin barriers, different films and membranes are being developed [5]. Unlike other materials, nanomaterials have unique physical (nanoscale size, high surface-to-volume ratio, diversity of morphology and structure, etc.), chemical (corrosion resistance, high reactivity, etc.), and biological properties (biocompatibility, low immunogenicity, etc.) which allow for nanomaterials to be widely used in tissue engineering [6]. With the increasing emphasis on environmental friendliness, nanomaterial templates are receiving a lot of attention and are considered one of the most promising techniques [7]. Moreover, the template allows for the structure to be reconstructed with the best possible reproducibility and plays the role of a skeleton, which can be used to develop various functions and adapt the created nanostructure in different areas [8,9]. In recent years, there has been a lot of interest in the potential application of nanopillars to membranes and films [10]. In fact, nanopillar surfaces have properties that make them more valuable than conventional planar surfaces in biological applications, such as medical therapy, tissue engineering, and antibacterial agents [11,12,13,14]. The nanopillars have three effects on surface biological processes: increase the surface area, increase cell adhesion and growth, and increase the ability to penetrate the cell [15].

One of the possibilities to fabricate nanopillars is the template method. Nanomaterials templates can be made using a variety of methods (additive manufacturing (3D printing) [16,17], lithography [18]), but most of them are relatively complex and expensive compared to the anodization process, which can be characterized by high efficiency, simplicity, low cost, and the notable qualities of the obtained nanostructures [19]. The anodization process in the fabrication of anodic aluminum oxide (AAO) nanoporous membranes is superior because structural changes can be obtained by controlling the electrochemical process conditions (type of electrolyte, electrolyte concentration, temperature, anodization time, anodizing potential, current density, etc.) during the fabrication process [20,21]. Furthermore, AAO nanoporous membranes have exceptional properties, such as a high thermal stability, mechanical hardness, and chemical inertness [22]. The use of the nanoporous AAO membrane as a template has several advantages over other types of templates, such as a nanometer-sized diameter and straight micropore length, low cost, and thermal stability over a wide temperature range without loss of porous structure, even after processing above 1000 °C; in addition, porous alumina can be used with a barrier layer on one side and a main porous oxide layer on the other side, or base materials can be removed on both sides to obtain a self-sustaining nanohole structure that can be used as a template [23,24,25].

Research has shown that chitosan is a promising candidate for the development of biocompatible, bactericidal, and bio-adhesive skin barriers and is already widely used in commercial wound dressings [10,26,27,28], hemostatic dressings [29] or drug delivery systems for wound healing [30]. At present, many types of commercial products based on chitosan (OneStop™ Bandage Rx, Hemcon^®^ Bandage PRO, HemCon ChitoDot^®^, ChitoGauze^®^ XR PRO (Tricol Biomedical, Portland, OR, USA)) are available for wound care [31]. To improve its properties, various derivatives of chitosan can be produced [32]. For example, the combination of chitosan and gelatin not only produces a biomatrix that is more durable, but also allows for the controlled release of biological agents and protects the wound from contaminating molecules [33]. The antimicrobial activity of chitosan and its potential for osteogenesis in stem cell culture environments were also presented by researchers [34]. Due to the less transferable nanostructure on the chitosan membrane, there are few studies on the micro/nanopatterning of chitosan membranes and films; therefore, two methods are commonly used: low-pressure, low-temperature nanoimprinting and solvent casting [35]. However, the low-pressure, low-temperature nanoimprinting method required careful control of the viscosity and the use of a nanoimprinter; therefore, the solvent casting method is the easiest and most-used method to transfer the nanostructure onto the chitosan membrane when the polymer solution is poured onto the patterned template [36]. When the bottom of the pores of the AAO template is closed, it is difficult to ensure the flow of the solution into the nanopores, so different chitosan derivatives could be used for this purpose [37], or the AAO template could be modified with surface energy-reducing agents [34].

Because chitosan membranes with nanopillars are promising in the creation of barriers for skin protection, it is important to develop fabrication technologies that involve controlling the geometry of nanopillars. This study presents the fabrication of a chitosan membrane with nanopillars using the nanoporous AAO membrane as a template. Because the solvent casting method was performed without high-frequency excitation in previous studies, the novelty of this study is that a free-standing chitosan membrane was prepared using a vibration-assisted solvent casting method and the influence of high-frequency excitation on the geometry of the nanopillars is analyzed. Moreover, in this study, the velocity of the chitosan solution into the closed nanopore was calculated mathematically. Therefore, to control the geometry of the nanopillars and expand the application of templates in membrane production, free-standing nanopillared chitosan membranes were produced, using 1 wt%, 2 wt% and 3 wt% concentrations of chitosan solutions to determine the influence on the formation of nanopillar using high-frequency excitation of 40 kHz for 5 s. Moreover, the effect of vibration on the surface area of the nanopillared chitosan membrane was also investigated. Furthermore, the velocity of the fluid flow into the nanopore, the height of the nanopillars, and the surface area of a single nanopillar were theoretically evaluated using COMSOL Multiphysics 6.0 software.

## 2. Materials and Methods

### 2.1. Fabrication of Nanoporous AAO Template

**Figure 1:**
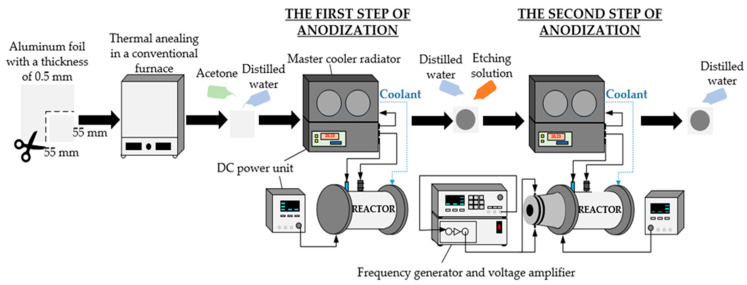
The synthesis route of nanoporous AAO template.

To control the geometry of the nanopillars and expand the application of templates in membrane fabrication, nanopillared chitosan membranes were fabricated using the nanoporous AAO templates. A facile and cost-effective two-step anodization process was used to fabricate the nanoporous AAO templates. The synthesis route of the nanoporous AAO template is shown in Figure 1.

**Figure 2:**
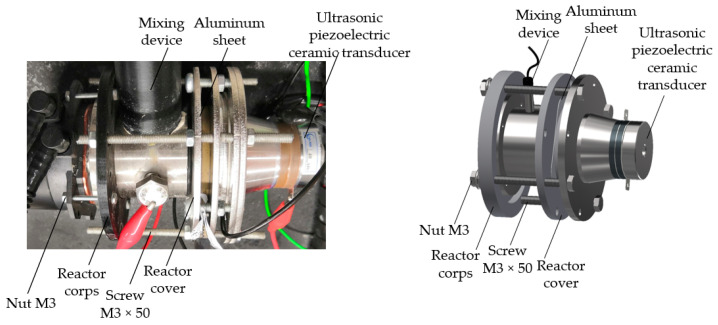
A novel design of the electrochemical reactor.

In this study, annealed aluminum foil of high purity (>99%) was used. First, the specimens of aluminum foil with a size of 55 × 55 × 0.5 mm were annealed at 400 °C for 4 h in a nitrogen environment in a conventional furnace. The samples were then cleaned with distilled water after being degreased with acetone. An electrochemical reactor was used to prepare the nanoporous AAO templates. The novel design of the electrochemical reactor is presented in Figure 2.

Using a 0.3 mol/L oxalic acid solution (H_2_C_2_O_4_) as the electrolyte, the two-step anodization process was conducted at a voltage of 60 V and a temperature of 5 °C. The initial anodization process took one hour. A solution of 3.5% concentrated phosphoric acid (H_3_PO_4_) and 2% concentrated chromium anhydride (CrO_3_) acid in water was used to etch the oxide layer. The chemical etching process was performed at 80 °C for 10 min. The second step of the anodization process was carried out in the electrochemical reactor at 60 V and 5 °C for 4 h after they had been cleaned with distilled water. During the second step of anodization, high-frequency excitation was used. More information on the anodization process using high-frequency excitation is given in [38]. Lastly, the produced nanoporous AAO templates were washed with distilled water and air-dried.

Additionally, using the Hitachi S–3400N (Hi-Tech Instruments, Bandar Bukit Puchong, Malaysia) scanning electron microscope (SEM), the geometry of the AAO pores was examined. By evaluating the pore diameters and interpore distances of the entire 45 mm diameter AAO membrane area and calculating the normal distribution of the pore diameters and interpore distances, the average values of the pore diameters and interpore distance were determined using the “ImageJ” data-processing program. Futhermore, the surface chemical composition of the AAO template was examined using Bruker Quad 5040 EDS energy dispersive X-ray spectroscopy.

### 2.2. Fabrication of Nanopillared Chitosan Membrane

**Figure 3:**
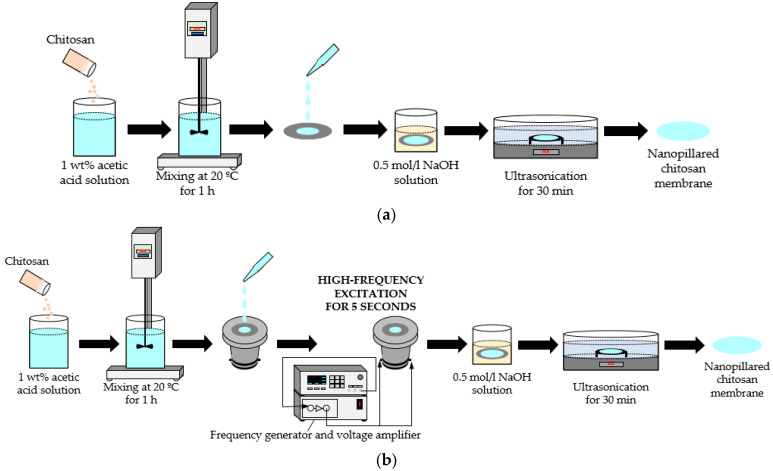
Fabrication of nanopillared chitosan membrane: (**a**) standard preparation procedure; (**b**) preparation procedure using high-frequency excitation.

1 wt%, 2 wt% and 3 wt% chitosan (high molecular weight, deacetylation degree >95%) were dissolved in 1 wt% acetic acid solution. The preparation procedure for the nanopillared chitosan membrane is shown in Figure 3.

The mixtures were stirred at 20 °C for 1 h, until the chitosan was completely dissolved. In the next step, the chitosan solution was added dropwise (casting temperature 20 °C) onto the prepared nanoporous AAO templates (diameter 45 mm) and dried at the temperature of 20 °C for 24 h. To dissolve the template, the samples were immersed in the 0.5 mol/L NaOH solution. Finally, the nanopillared chitosan membranes were cleaned by ultrasonication in distilled water. The geometry of chitosan nanopillars was examined using SEM.

**Figure 4:**
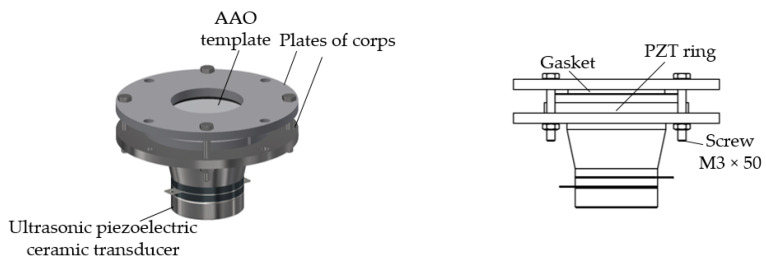
The device for the high-frequency excitation of chitosan solution and AAO template.

To determine the influence of vibration on the geometry of the nanopillars, after dropping chitosan onto the nanoporous AAO template, the chitosan, together with the AAO template, was vibrated at the frequency of 40 kHz for 5 s, and then all procedures were conducted as usual. The device for chitosan and AAO template vibrations is presented in Figure 4.

The structures of nanopillars are primarily responsible for their unique properties. Numerous vertically aligned nanopillars considerably increase the surface area of an original surface without altering the original substrate’s size. Formula (1) could be used to determine the surface area (S) of the nanopillared surface [16]:

S = S_0_ + n (2πrl) (1)

where S_0_ is the area of the original flat surface, r is the radius of each nanopillar, l is the height of the nanopillars, and n is the number of nanopillars on the flat surface.

### 2.3. Simulation Method and Conditions of Vibration Process

Due to recent advances in the production of microfluidic systems, acoustics are used to control microparticles, living cells, and other particles [39]. Using acoustics, an acoustic streaming flow is created in the microchannels, which also affects the particles with a viscous drag force [40]. The forces of viscous drag and acoustic radiation control the particle trajectories [41]. Due to the non-linear terms in the governing equations, the acoustic radiation force is the effect of the transfer of momentum from the acoustic field to the particles [42]. As a result, the acoustic radiation force acts as a net force on the particles [43].

Acoustic streaming is a term for the net time-averaged flow that results from harmonic disruption of the flow due to non-linear factors in the Navier–Stokes equations [44]. A second-order (nonlinear) acoustic effect is acoustic streaming [45]. The viscous drag force on the particles is influenced by the acoustic streaming and the balance between the acoustic radiation force and the viscous drag force (from the streaming flow) dominates the trajectory of the particles [46].

In this study, the theoretical model was a multiphysics task that included three main stages. The first stage was related to the determination of the first-order acoustic field in the domain, the second included the acoustic streaming flow in the domain, and the third stage was related to the flow of the fluid (also evaluating the streaming flow) inside the pore.

Firstly, the first-order acoustics field was solved by the Thermoviscous Acoustics and Frequency Domain interface in the model [47]. The acoustic boundary layer was determined, and the streaming flow in this layer was obtained. Then, the relevant source terms from the first-order fields were added to the Laminar flow interface to solve the second-order time-averaged net flow. Pressure Acoustics, Frequency Domain, and Thermoviscous Boundary Layer Impedance were used to account for the damping in the thin viscous boundary layers.

The steady-state streaming flow is described by the formulas:

0 = −∇ · (ρ_0_ u_2_) − ∇ · (<ρ_1_ u_1_>) (2)

0 = ∇ · σ_2_ − ∇ · (ρ_0_ <u_1_ u_1_^T^>) (3)

where ρ is the density (kg/m^3^), u is the fluid velocity (m/s), T is the temperature (K), <…> is the time averaging and subscript 2 is the streaming flow variables.

Formula (4), governing the transport and reinitialization of ϕ, can be written as [48]:

∂ϕ/∂t + u · ∇ ϕ = γ∇ · (ε∇ ϕ − ϕ (1 − ϕ) (∇ϕ/|∇ϕ|))(4)

where ϕ is the isocontour (determines the position of the interface), t is the time (s), γ is the reinitialization parameter (m/s), which determines the amount of reinitialization, and ε is the reinitialization parameter (m), which determines the layer thickness surrounding the interface where ϕ goes from zero to one).

An interface thickness can be described by the following formula:

ε = h_c_/2 (5)

where h_c_ is the characteristic mesh size in the area passed by the interface.

The parameter ϕ is also used to calculate the density and dynamic viscosity:

ρ = ρ_ch_ + (ρ_a_ − ρ_ch_) ϕ (6)

µ = µ_ch_ + (µ_a_ − µ_ch_) ϕ (7)

where ρ_ch_, µ_ch_, ρ_a_, µ_a_ are the constants of density and viscosity of chitosan solution and air, respectively.

The incompressible Navier–Stokes equations, which include surface tension, regulate the transfer of mass and momentum in the Laminar Two-Phase Flow and Level Set interface:

ρ (∂u/∂t + u · ∇u) = −∇p + ∇ · µ (∇u + ∇u^T^) + ρg + F_st_ (8)

∇ · u = 0(9)

where p is the pressure (Pa), µ is the viscosity (Pa·s), ρg is the gravity (m/s^2^), F_st_ is the surface tension force components.

The surface tension force can be described by the following formula:

F_st_ = ∇ · T = ∇ · [σ {I + (−nn^T^)} δ] (10)

where σ is the surface tension coefficient, I is the identity of the matrix, n is the interface unit normal, δ is a Dirac delta function (nonzero only at the fluid interface).

The interface normal could be calculated from the following formula:

n = ∇ϕ/|∇ϕ|(11)

The parameter ϕ can be used to approximate the delta function:

δ = 6 |ϕ (1 − ϕ)| |∇ϕ|(12)

**Figure 5:**
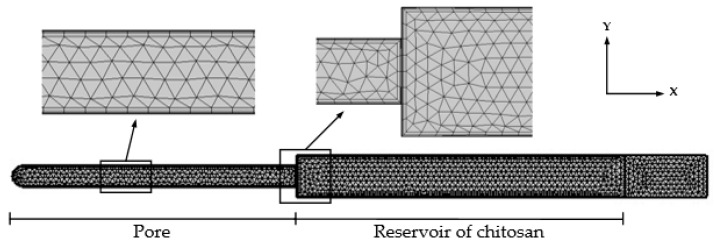
The finite element mesh of the simulation model.

In this study, COMSOL Multiphysics 6.0 software was used to simulate the behaviour of fluids in the nanopore. The deposition of the chitosan solution on the porous AAO structure and the flow of liquid into the pores was simulated using high frequencies. The mathematical model was composed of one pore with a diameter of 100 nm and a chitosan reservoir. The model was meshed by free triangular elements with a boundary layer inside the structure. The finite element mesh is shown in Figure 5.

The inner wall of the AAO nanopore was determined to be solid surfaces with no-slip and isothermal boundary conditions. The walls of the chitosan reservoir were determined to be a wetted wall with a navier slip condition. The model was composed of 4213 domain elements and 453 boundary elements. Movement in the Y direction was constrained, and periodic movements occurred in the X direction. The main model parameters are presented in Table 1.

Thermoviscous acoustics, laminar flow, and level set physics were applied to the entire model. In the multiphysics discipline, the two-phase flow was selected. Here, the laminar flow was selected as the fluid flow and the level set as the moving interface. The temperature of the model was set at room temperature, at 20 °C.

## 3. Results and Discussion

### 3.1. Investigation of Porous AAO Template

In this section, the fabricated nanoporous AAO template is described. To produce nanopillared chitosan membranes, nine identical nanoporous AAO templates were fabricated. For the pore diameter, the distance between pores and thickness analysis, the “ImageJ” data-processing program was used. The average values of the pore diameters and interpore distance were obtained by evaluating the pore diameters and interpore distances of the entire 45 mm diameter AAO membrane area, and the normal distribution of pore diameters and interpore distances were calculated. The results of the geometry of the nanoporous AAO template are presented in Table 2.

**Figure 6:**
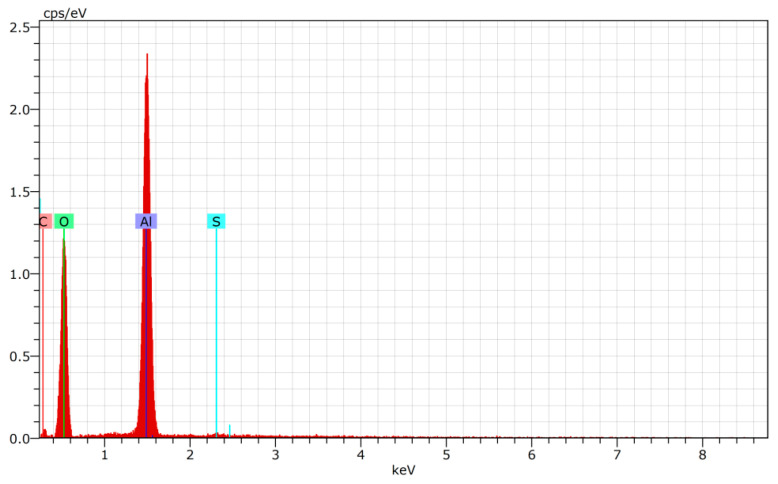
The EDS curve of AAO template.

To determine the chemical elements and their quantity in the porous AAO template, EDS analysis was used. The elements determined during EDS analysis are presented in Table 3 and Figure 6.

According to Table 3 and Figure 6, two elements (aluminum and oxygen) predominated. This confirmed the formation of A_2_O_3_. Sulfur and carbon were identified as impurities due to amorphous A_2_O_3_.

### 3.2. Theoretical Results of the Flow of Different Concentrations of Chitosan Solution into the AAO Nanopores

**Figure 7:**
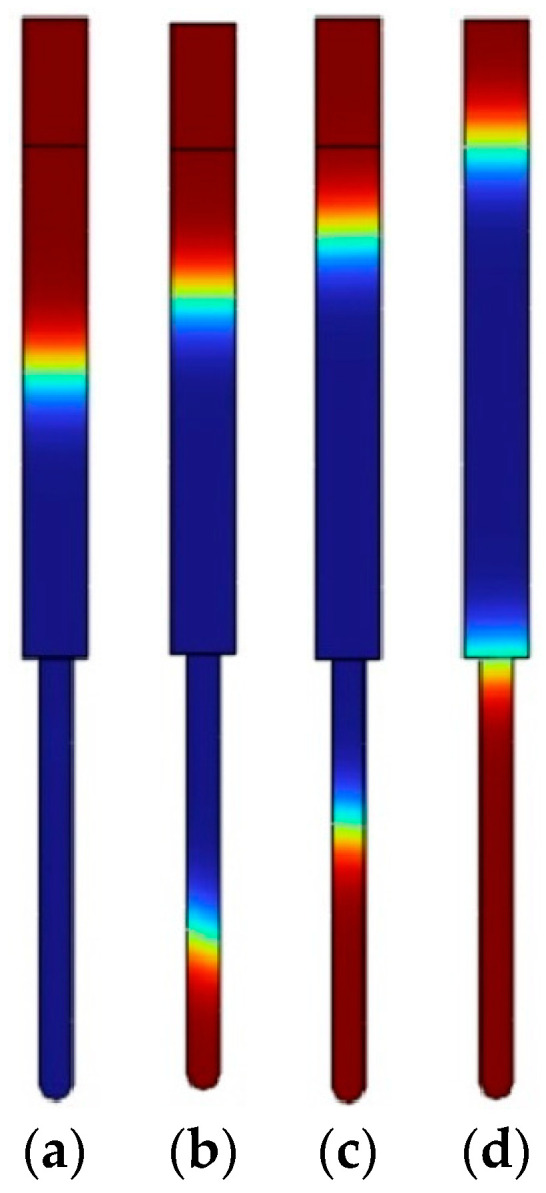
Flow of different concentrations of chitosan solution into the nanopore using the high-frequency excitation of 40 kHz in 4 s: (**a**) 1 wt% chitosan solution; (**b**) 2 wt% chitosan solution; (**c**) 3 wt% chitosan solution; (**d**) any concentration of chitosan solution without high-frequency excitation. The blue represents chitosan solution, and red represents the air.

The results of the first and second simulations showed the distribution of acoustic velocity and streaming velocity. The acoustic velocity showed the propagation of the sound waves through the air in the pore and the chitosan solution. Affected by the streaming velocity, the chitosan solution gained enough energy to flow into the pore. The flow of chitosan solutions of different concentrations into the pore was calculated during the last simulation, and the volume fraction of fluid was obtained. The resulting images of the simulations are presented in Figure 7.

The results related to the velocity, the height of the nanopillars, and the surface area of a single nanopillar are presented in Table 4.

Using the high-frequency excitation of 40 kHz in 4 s, the velocity of the 1 wt% chitosan solution was 250 nm/s, while the velocity of the 2 wt% chitosan solution was 169 nm/s under the same conditions. The speed of the 3 wt% chitosan solution was the lowest and reached 94 nm/s. However, when high-frequency excitation was not used, the flow of the chitosan solution into the closed nanopore was not ensured and a flat surface was obtained. Using high-frequency excitation, the results showed that as the concentration of the solution decreased, the height of the nanopillars increased, which led to an increase in the surface area. When the concentration varied from 3 wt% to 1 wt%, the surface area of the single nanopillar increased more than twice under the same conditions. The results led to an increase in the surface area of the chitosan membrane compared to a flat chitosan membrane. Furthermore, by changing the concentration of the chitosan solution during production, the required surface area can be calculated and obtained according to the surface area shown in Formula (1).

### 3.3. Investigation of Nanopillared Chitosan Membrane

**Figure 8:**
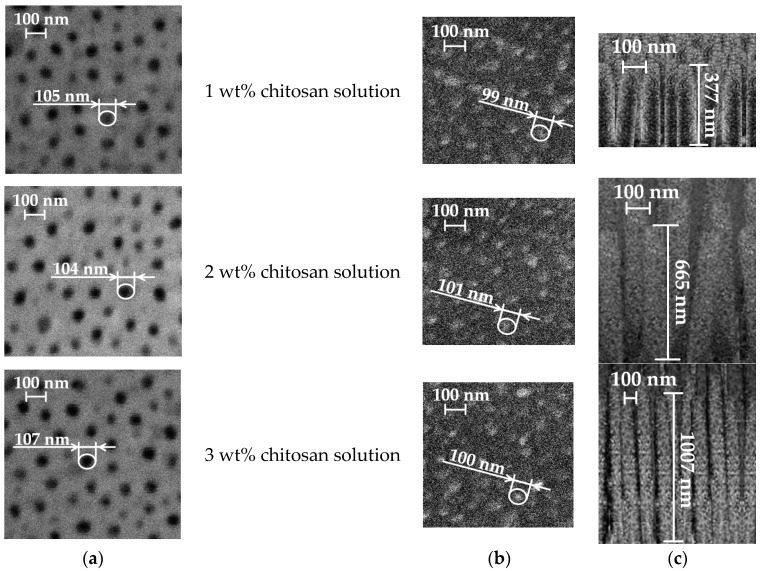
SEM images of: (**a**) nanoporous AAO template; (**b**) chitosan membrane; (**c**) nanopillar of chitosan membrane (cross-section).

The nine free-standing chitosan membranes (three membranes of each concentration) were prepared by the vibration-assisted solvent casting method. Nanopillared chitosan membranes were experimentally prepared from different concentrations of chitosan solutions to determine their influence on the formation of nanopillars using high-frequency excitation. SEM images of the nanoporous AAO templates, chitosan membranes, and nanopillars are presented in Figure 8.

The experimental results of chitosan membranes using 1 wt%, 2 wt%, and 3 wt% chitosan solutions are presented in Table 5.

In all cases where a high-frequency excitation of 40 kHz was used during the solvent casting method, nanopillared chitosan membranes were obtained. Depending on the concentration of the prepared chitosan solution, nanopillars of different heights were obtained. It can be assumed that the diameter of the nanopillars did not depend on the concentration, and the obtained nanopillar diameters were 100 ± 10 nm in all cases. However, changes in the height of the nanopillars were observed.

To obtain the height of nanopillars as in the theoretical calculations, high frequency was applied for 5 s. Experimental liquid flow velocities were obtained, at 201 nm/s, 133 nm/s and 75 nm/s, respectively, for concentrations of 1 wt%, 2 wt% and 3 wt%. With the experimental velocities of chitosan solutions into the pore and the concentration of the chitosan solution, the heights of the nanopillars could be controlled by high-frequency excitation. It was experimentally found that high-frequency excitation ensured fluid flow into the nanoporous AAO template, and the heights of the nanopillars could be controlled by changing the concentration of the chitosan solution.

**Figure 9:**
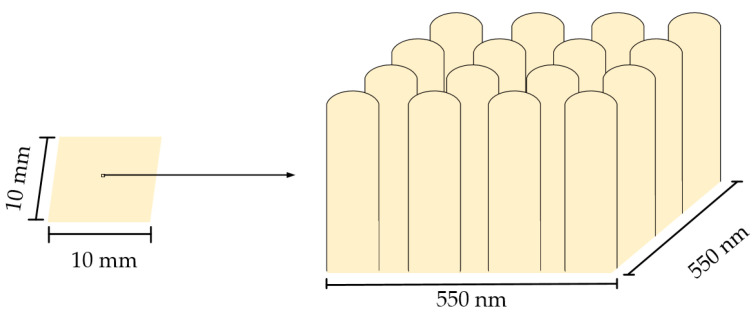
Free-standing chitosan membrane.

As a result of the changing height of the nanopillars, the surface area of the membrane changed, and the research contributes to the wider application of chitosan membranes as an artificial skin barrier. To develop artificial skin barriers, surface area calculations were performed for the free-standing chitosan membrane with a size of 10 × 10 mm (Figure 9).

According to Formula (1), the surface areas were calculated when the height of the nanopillars was 377, 665 and 1007 nm. In all cases, the area of the original flat surface was 1 cm^2^, the number of nanopillars was 4.4 · 109, and the radius of each nanopillar was 50 nm. Based on the experimental data, the values of the calculated surface area are given in Table 5. Compared to the original flat surface area, the surface area of the nanopillared chitosan membrane could be increased by 6, 10, and 15 times when chitosan solution with different concentrations was poured onto the nanoporous AAO template using a high-frequency excitation of 40 kHz for 5 s.

## 4. Conclusions

In this study, free-standing nanopillared chitosan membrane was fabricated using the improved solvent casting method on the nanoporous AAO template. The high-frequency excitation of 40 kHz was used during the solvent casting method to improve the flow of liquid into the nanopore. Nanopillared chitosan membranes were successfully fabricated using 1 wt%, 2 wt% and 3 wt% solutions of chitosan in acetic acid. SEM images confirmed the formation of AAO nanopores and chitosan nanopillars. Three types of chitosan membranes were experimentally obtained when the height of the nanopillars was 1007, 665, and 377 nm. From the obtained heights of the nanopillars, according to the surface area formula, the surface areas of the free-standing nanopillared chitosan membranes (with a size of 10 × 10 mm) were calculated, which were 15.05, 10.28 and 6.26 cm^2^. Compared to the flat membrane surface, the nanopillared surface area was increased by 15, 10, and 6 times. Furthermore, the experimental velocities of chitosan solution into the pore were 201, 133 and 75 nm/s, determined at concentrations of 1 wt%, 2 wt% and 3 wt% chitosan solution, respectively. The experimentally determined liquid flow velocities into the nanopore make it possible to form nanopillars of the desired height, which leads to precise control of the surface area. Due to the easily controlled surface area, these studies contribute to the development of artificial skin barriers for commercial use.

## Figures and Tables

**Table 1:** The model parameters.

Parameter	Symbol	Inscription	Value
Frequency	f0	40 [kHz]	40.000 Hz
Ambient temperature	T0	20 [°C]	293.15 K
Ambient pressure	p0	1 [atm]	1.0133 × 10^5^ Pa
Study angular frequency	omega0	2 × pi × f0	2.5132 × 10^5^ Hz
Channel cross-section width	W	100 [nm]	1 × 10^−7^ m
Channel cross-section height	H	1000 [nm]	1 × 10^−6^ m
Wall displacement	d0	100 [nm]	1× 10^−7^ m
Time	t	4 [s]	4 s

**Table 2:** Average pore diameter and interpore distance of porous AAO templates.

Parameter	Value	Image
Pore diameter, nm	100 ± 10	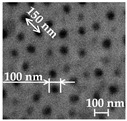
Interpore distance, nm	150 ± 10	
Thickness, µm	45 ± 1	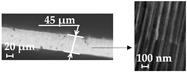

**Table 3:** Chemical composition of AAO template.

	Element			
	Aluminum	Oxygen	Carbon	Sulfur
Normalised concentration, wt%	44.90	53.38	1.24	0.48
Atomic concentration, at%	32.51	65.18	2.02	0.29
Error, %	2.4	7.8	0.5	0.1

**Table 4:** Simulation results of chitosan solution with concentrations of 1 wt%, 2 wt% and 3 wt%.

Parameter	1 wt%	2 wt%	3 wt%
Velocity, nm/s	250	169	94
Height of nanopillars, nm	1000	675	375
Surface area of a single nanopillar, µm^2^	0.314	0.212	0.118

**Table 5:** Experimental results of chitosan membrane.

Parameter	1 wt%	2 wt%	3 wt%
Diameter of nanopillars, nm	99 ± 10	101 ± 10	100 ± 10
Height of nanopillars, nm	1007 ± 10	665 ± 10	377 ± 10
Average surface area of a single nanopillar, µm^2^	0.313	0.211	0.118
Calculated experimental velocity, nm/s	201	133	75
Calculated surface area, cm^2^	15.05	10.28	6.26

## Data Availability

Data sharing is not applicable.
